# Blood-Based SOX2-Promoter Methylation in Relation to Exercise and PM_2.5_ Exposure among Taiwanese Adults

**DOI:** 10.3390/cancers12020504

**Published:** 2020-02-21

**Authors:** Chun-Lang Su, Disline Manli Tantoh, Ying-Hsiang Chou, Lee Wang, Chien-Chang Ho, Pei-Hsin Chen, Kuan-Jung Lee, Oswald Ndi Nfor, Shu-Yi Hsu, Wen-Miin Liang, Yung-Po Liaw

**Affiliations:** 1 Department of Public Health and Institute of Public Health, Chung Shan Medical University, Taichung 40201, Taiwan; s901055@gmail.com (C.-L.S.); tantohdisline@yahoo.com (D.M.T.); wl@csmu.edu.tw (L.W.); c0701chen@gmail.com (P.-H.C.); jasminemachi@gmail.com (K.-J.L.); nforoswald2@yahoo.com (O.N.N.); sui0209@gmail.com (S.-Y.H.); 2 School of Medicine, Chung Shan Medical University, Taichung 40201, Taiwan; 3 Department of Physical Medicine and Rehabilitation, Yuan Sheng Hospital, Yuanlin 510, Taiwan; 4 Department of Medical Imaging, Chung Shan Medical University Hospital, Taichung 40201, Taiwan; 5 Department of Radiation Oncology, Chung Shan Medical University Hospital, Taichung 40201, Taiwan; hideka.chou@gmail.com; 6 School of Medical Imaging and Radiological Sciences, Chung Shan Medical University, Taichung 40201, Taiwan; 7 Institute of Medicine, Chung Shan Medical University, Taichung 40201, Taiwan; 8 Department of Physical Education, Fu Jen Catholic University, New Taipei 24205, Taiwan; ccho1980@gmail.com; 9 Department of Health Services Administration, China Medical University, Taichung 40402, Taiwan; wmliang@mail.cmu.edu.tw

**Keywords:** exercise, PM_2.5_, SOX2, methylation, Taiwan Biobank

## Abstract

Increased ventilation during exercise in polluted areas could trigger airway inflammation. We evaluated blood DNA methylation of the SOX2-promoter region in relation to exercise and PM_2.5_ in Taiwanese adults. Data of 948 participants aged 30–70 years were retrieved from the Taiwan Biobank Database (2008–2015) and the Air Quality Monitoring Database (2006–2011). PM_2.5_ was positively associated with SOX2-promoter methylation (β = 0.000216; *p* < 0.0001). The interaction between PM_2.5_ and exercise on SOX2-promoter methylation was significant (*p* = 0.0146). After stratification by exercise habits, PM_2.5_ was positively associated with SOX2 methylation in only individuals who did regular exercise (β = 0.0003490; *p* < 0.0001). After stratification by exercise habits and residential areas, SOX2-promoter methylation levels in those who lived in the southern area were higher for both the regular exercise (β = 0.00272; *p* = 0.0172) and no regular exercise groups (β = 0.002610 and *p* = 0.0162). SOX2-promoter methylation levels in those who lived in the northern area and did regular exercise were lower; β = -0.00314 (*p* = 0.0036). In conclusion, PM_2.5_ was positively associated with SOX2-promoter methylation in participants who did regular exercise. Living in the southern area was positively associated with SOX2-promoter methylation regardless of exercise habits.

## 1. Introduction

Exercise may improve lung function and quality of life [1]. It is associated with a lower risk of cancer [2,3,4] and cancer-specific, as well as all-cause mortality [5,6]. A well designed and ideal exercise regimen could serve as a low-cost supplementary therapy that could relieve symptoms and improve outcomes in chronic lung disease and cancer, particularly lung cancer [3,4,7,8]. However, in polluted areas, increased ventilation during exercise could ease the deposition of air pollutants in the lungs [9,10,11], resulting in an increased risk of airway inflammation and reduced lung function [12,13]. For instance, the risks associated with short-term exposure to traffic pollution outweighed the cardiopulmonary benefits associated with exercise in individuals with ischemic heart disease and chronic obstructive pulmonary disease (COPD) [14].

Air pollutants, particularly particulate matter (PM), are classified as group 1 carcinogens [15] and are a great threat to global environmental health [16,17]. Some adverse effects of exposure to PM are inflammation, cytotoxicity due to oxidative stress, genotoxicity, and subsequent DNA damage and carcinogenicity [18,19]. Fine PM, that is, PM whose diameter is less than 2.5 μm (PM_2.5_) is one of the well-established risk factors for lung cancer [20,21,22,23,24,25]. It accounted for 7.6% of global deaths in 2015 and was the fifth leading cause of death [26].

Epigenetic changes could explain the mechanism behind the health impacts of air pollution and have been proposed as tools that could determine the systemic effects of air pollution [27,28]. For instance, toxic components of PM_2.5_ like arsenic, polycyclic aromatic hydrocarbon (PAH), chromium, cadmium, lead, and mercury cause DNA methylation, the most studied and well-understood epigenetic process [29,30,31,32,33]. The modulation of promoter DNA methylation by PM_2.5_ is an essential mechanism that alters the expression of genes [19]. DNA methylation changes, alongside inflammation, apoptosis, autophagy, oxidative stress, among others, are some of the main mechanisms underlying the role of PM_2.5_ in lung cancer development [34].

The SOX2 gene is an embryonic stem cell transcription factor located on chromosome 3 [35,36]. SOX2 plays a crucial role in induced cellular reprogramming and regulates pluripotency or self-renewal in embryonic stem cells [35,36,37,38,39]. It is associated with cancer-promoting processes like proliferation, evasion of apoptosis, cell invasion, and metastasis [35,40]. It is considered to be a molecular marker for the diagnosis of cancer especially lung squamous cell carcinoma [41,42].

Endurance exercise helps in the expansion of developmentally early stem cells including SOX2 [43]. However, to our knowledge, studies on SOX2 methylation and exercise are limited. Even though both air pollution [29,30,31,32,33] and exercise [44,45] are associated with DNA methylation, the relationship of exercise and PM_2.5_ with SOX2-promoter methylation has not been extensively explored. To date, only our previous study assessed the association between PM_2.5_ and SOX2 methylation in Taiwanese adults [46]. Moreover, the relationship between exercise and exposure to air pollution is still controversial. While some studies have reported that the benefits of exercising outweigh the risks associated with air pollution during exercise [47,48], others have reported the opposite [12,13,14]. In addition, the mechanism behind the relationship between exercise and PM_2.5_ exposure and their associated epigenetic outcomes are yet to be elucidated. Given the crucial role of PM_2.5_ and exercise in DNA methylation and cancer, we conducted this study to assess the association of exercise and PM_2.5_ with SOX2-promoter methylation in Taiwanese adults aged 30–70 years.

## 2. Results

There were 948 participants, including 488 men and 460 women. Among these participants, 414 (43.67%) did regular exercise while 534 (56.32%) did not do regular exercise. The mean (± SE) SOX2-promoter methylation level in those who did regular exercise was 0.1635 ± 0.000657. This mean methylation level was significantly different (*p* < 0.0001) from that (0.1596 ± 0.00055600) in those who did not do regular exercise (Table 1). The mean annual concentrations of PM_2.5_ were 26.557, 30.055, 36.907, and 40.683 μg/m^3^ in the northern, north-central, central, and southern areas, respectively (Table 1).

Exercise was not significantly associated with SOX2-promoter methylation. However, PM_2.5_ was significantly associated with higher levels of SOX2-promoter methylation (β = 0.000216; *p* < 0.0001). The interaction between exercise and PM_2.5_ on SOX2-promoter methylation was significant (*p* = 0.0146), as shown in Table 2.

After stratification by exercise habits (Table 3), PM_2.5_ was significantly associated with higher SOX2 methylation levels only in individuals who did regular exercise (β = 0.0003490; *p* < 0.0001). After further stratification by exercise habits and residential area (Table 4), SOX2-promoter methylation levels in those who lived in the southern area were higher in both the regular exercise (β = 0.00272; *p* = 0.0172) and no regular exercise groups (β = 0.002610 and *p* = 0.0162). On the other hand, SOX2-promoter methylation levels in those who lived in the northern area and did regular exercise were lower; β = −0.00314 and *p* = 0.0036 (Table 4).

## 3. Discussion

In this study using DNA extracted from blood of men and women aged 30–70 years, PM_2.5_ was positively associated with SOX2-promoter methylation. However, exercise was not significantly associated with SOX2-promoter methylation. The interaction between PM_2.5_ and exercise on SOX2-promoter methylation was significant. After stratifying the participants by exercise habits, PM_2.5_ was positively associated with SOX2-promoter methylation. However, the association was significant only in those who did regular exercise. After further stratification of participants based on exercise habits and residential areas, living in the southern area was positively associated with SOX2-promoter methylation, irrespective of exercise habits. This implies that the influence of PM_2.5_ on SOX2 might be greater than that of exercise. However, the underlying mechanism cannot be clearly stated. Pollution affects DNA methylation by disrupting DNA methyltransferase (DNMT) activities and altering DNA methylation substrates like S-adenosine methionine [49,50]. For instance, air pollutants could alter the efficiency of DNMTs or decrease their expression levels [29,50,51].

It is worth stating that the relationship between air pollution and exercise is not consistent. For instance, the benefits of exercise on asthma and COPD outweighed the adverse effects of pollution exposure during exercise [47]. In addition, at a lower concentration (22 μg/m^3^) of PM_2.5,_ the health benefits of physical activity were greater than the risks of exposure to PM_2.5_. However, at a higher concentration (100 μg/m^3^) of PM_2.5_, the harm exceeded the health benefits [48]. In the same manner, the risks of exposure to traffic pollution outweighed the health benefits associated with exercise in individuals with ischemic heart disease and COPD [14]. In addition, exposure to higher levels of air pollution was associated with more adverse effects in susceptible individuals [9]. In our previous study, PM_2.5_ was positively associated with SOX2-promoter methylation in non-smoking Taiwanese adults [46]. In this study, SOX2 methylation levels were higher in individuals who resided in the southern area of Taiwan (which have higher levels of PM_2.5_), regardless of exercise habits. Since it is important to adjust each predictor’s *p*-value, we also analyzed our data using the penalized linear regression model. The beta coefficients obtained by penalized linear regression were similar to those obtained by general linear regression. However, BMI was not retained in the penalized regression analysis (Tables S1–S3). To our knowledge, the association between exercise and SOX2-promoter methylation has not been widely explored.

Endurance exercise was associated with the expansion of developmentally early stem cell genes including SOX2 [43]. Moreover, cancer stem cell markers like SOX2 and OCT4 and related levels of microRNAs are believed to be potential biomarkers for predicting carcinogenicity resulting from exposure to PM_2.5_ [25]. PM_2.5_ exposure was associated with the induction of cancer stem cell properties, marked by upregulation of mRNA levels of pluripotency-maintaining genes including SOX2 and OCT4 and subsequent increase in the risk of lung cancer [25,52]. The activation of the Notch pathway is believed to be one of the mechanisms underlying the PM_2.5_-induced cancer stem cell properties [52].

SOX2 methylation is associated with several types of cancer, including small-cell lung cancer (SCLC), squamous cell carcinoma (SCC), glioblastoma, endometrial, and breast cancer [35,36,37,38,39,42]. Moreover, SOX2 levels influence cell fate decisions and are crucial determining factors in the proliferation of both normal and cancer cells [53]. For example, anomalous levels (either too much or too little) are believed to adversely influence cell fate decisions during the process of development in normal cells [53]. SOX2 overexpression is thought of as a major contributor in maintaining the antiapoptotic and tumorigenic properties of cancer stem cells [54,55]. In glioblastoma, SOX2 promoter hypomethylation was associated with SOX2 overexpression [56]. In non-small cell lung cancer (NSCLC), the amplification and subsequent overexpression of the SOX2 gene were common in both squamous cell carcinomas and adenocarcinomas (with higher expressions in squamous cell carcinomas compared to adenocarcinomas) [42,57,58].

SOX2 amplification and overexpression have also been reported as independent poor prognostic predictors of stage I lung adenocarcinoma [59]. Notwithstanding, in non-small cell lung carcinoma (NSCLC), SOX2 overexpression was associated with a better prognostic course in only squamous cell carcinoma [58,60] and both squamous cell carcinoma and adenocarcinoma [42,57]. Furthermore, the absence of SOX2 expression was associated with poor esophageal and hypopharyngeal cancer prognosis [39,61]. Since SOX2 overexpression could stimulate cancer enhancing processes [54,55], it remains unclear why the loss of SOX2 expression is associated with malignant clinical phenotypes. It has been suggested that SOX2-silenced cancer cells have the potential to escape cell-cycle arrest and become resistant to apoptosis, thereby enhancing carcinogenesis [39].

Even though SOX2 methylation influences gene expression [39,62], we did not evaluate the correlation between SOX2-promoter methylation and SOX2 gene expression. This serves as the limitation of our study.

## 4. Materials and Methods

### 4.1. Study Participants

A total of 948 individuals (488 men and 460 women) between 30 and 70 years were included in the current study. Self-reported data, including age, sex, residential address, exposure to second-hand smoke (SHS), exercise, cigarette smoking, and alcohol drinking, as well as measured data, including body mass index (BMI) and SOX2 methylation levels, were retrieved from the Taiwan biobank database (2008–2015). This data source has been previously described [46,63,64]. The Taiwan Biobank participants are strictly Taiwanese who have never been clinically diagnosed with cancer [46,63,64]. Informed consents were obtained from participants during the recruitment phase of the biobank project. This study was approved by the Chung Shan Medical University Institutional Review Board (CS2-17070).

### 4.2. Variable Assessments

#### 4.2.1. DNA Methylation

Pure DNA samples from whole blood DNA were treated with sodium bisulfite using the EZ DNA Methylation Kit (Zymo Research, CA, USA) and DNA methylation was determined using the Infinium^®^ MethylationEPIC BeadChipEPIC array (Illumina Inc, San Diego, CA, USA). The Infinium^®^ MethylationEPIC BeadChipEPIC array measures methylation at more than 850,000 CpG sites and contains about 90% of the sites that are covered by the HumanMethylation450 BeadChip [65,66]. Quality control measures included (1) subtraction of background signals; (2) removal of probes with bead counts < 3 and probes with poor detection (*p*-value > 0.05); (3) removal of outliers using the median absolute deviation method; and (4) correction for dye-bias across batches by normalization. Methylation levels were calculated and expressed as beta-values (β). The beta-values range between 0 and 1 and are calculated as β = M/M + U. M represents the methylated intensity while U represents the unmethylated intensity.

In the current study, the mean beta-values from 24 CpG sites located in the promoter region of the SOX2 gene were used in our final analysis. The sites include cg00666105, cg01023203, cg01340005, cg02573703, cg04948892, cg05664581, cg07747133, cg08062338, cg08464053, cg09530873, cg11129008, cg11142406, cg12930100, cg14783675, cg15106134, cg17051733, cg18148179, cg19258425, cg20106776, cg22530053, cg24513480, cg24782772, cg25933341, and cg27331851.

#### 4.2.2. PM_2.5_ Pollution

Personal exposure to PM_2.5_ was estimated based on participants’ residential addresses. That is, PM_2.5_ concentrations from air monitoring stations where participants lived for at least three months were used as a proxy for personal exposure to PM_2.5_. The areas where participants lived were grouped into 4: the northern area (Taipei and New Taipei City), the north-central area (Hsinchu City, Taoyuan, Hsinchu, and Miaoli County), the central area (Taichung City, Changhua, Nantou, and Yunlin County), and southern area (Chiayi City, Chiayi, and Tainan County). The average PM_2.5_ concentrations (2006–2011) from these areas were retrieved from the Air Quality Monitoring Database (AQMD) of the Environmental Protection Administration, Taiwan.

#### 4.2.3. Exercise and Other Variables

Participants were categorized into two groups (regular and non-regular exercise) based on self-reported weekly exercise frequency. Activities considered as exercise included swimming, rope jumping, gymnastics, weight training, biking, jogging, hiking, yoga, aerobic dance, strolling, Chinese martial arts, “Qigong”, “Taijiquan”, hula hoop, table tennis, basketball, tennis, golf, soccer, badminton, and other ball games. Regular exercise was defined as engaging in one or more of the above activities over 30 minutes at least three times per week.

Cigarette smoking and alcohol drinking habits were categorized into three groups (never, former, and current) and exposure to second-hand smoke was grouped into two (yes and no). The definition of smoking and drinking habits, as well as exposure to second-hand smoke, have been previously provided [46,63].

#### 4.2.4. Statistical Analysis

Data were managed and analyzed with the SAS software, version 9.4 (SAS Institute, Cary, NC, USA). Categorical variables like sex, smoking, and drinking habits between the two exercise groups were compared using the Chi-squared test while continuous variables like SOX2 beta values, age, and BMI were compared using the *t*-Test. Categorical and continuous variables were presented in percentages (%) and mean ± standard error (SE), respectively. Multivariate linear regression models were used to determine the association of PM_2.5_ and exercise with SOX2-promoter methylation. Adjustments were made for covariates including sex, age, BMI, cigarette smoking, alcohol drinking, and exposure to second-hand smoke. Potential confounding due to cell-type heterogeneity was corrected using the Reference-Free Adjustment for Cell-Type composition (ReFACTor) method [67].

## 5. Conclusions

In conclusion, PM_2.5_ was positively associated with SOX2-promoter methylation and the interaction between PM_2.5_ and exercise on SOX2-promoter methylation was significant. After stratification by exercise habits, PM_2.5_ was positively associated with SOX2-promoter methylation in participants who did regular exercise. Based on exercise habits and residential areas, living in the southern area was associated with higher levels of SOX2-promoter methylation in participants regardless of the exercise habits. Even though the underlying mechanisms cannot be clearly stated, the findings imply that PM_2.5_ might influence SOX2 methylation more than exercise. Since SOX2 hypermethylation is associated with cancer development, taking exercise in heavily polluted areas may not be very helpful and therefore should be minimized. However, exercise is associated with several health benefits. Therefore, the air quality in the heavily polluted areas should be improved. Further studies, especially on mice, are recommended to support our findings.

## Figures and Tables

**Table 1:** Basic characteristics of the study participants stratified by exercise habits.

Variable	No Exercise	Exercise	*p*-Value
	(*n* = 534)	(*n* = 414)	
SOX2-promoter methylation (beta-value)	0.159600 ± 0.000556	0.163500 ± 0.000657	<0.0001 *
Residential area/mean PM_2.5_ in μg/m^3^ (%)			0.7951
Northern/26.557	186(34.83)	144(34.78)	
North-Central/30.055	92(17.23)	69(16.67)	
Central/36.907	111(20.79)	78(18.84)	
Southern/40.683	145(27.15)	123(29.71)	
Sex (%)			0.6106
Women	263(49.25)	197(47.58)	
Men	271(50.75)	217(52.42)	
Age (years)	46.1105 ± 0.4613	54.4010 ± 0.4969	<0.0001 *
BMI (Kg/m^2^)	24.3257 ± 0.1673	24.3130 ± 0.1549	0.9557
Cigarette smoking status (%)			0.0143
Never	397(74.34)	311(75.12)	
Former	66(12.36)	69(16.67)	
Current	71(13.30)	34(8.21)	
Second-hand smoke exposure (%)			0.0032 *
No	457(85.58)	380(91.79)	
Yes	77(14.42)	34(8.21)	
Alcohol drinking status (%)			0.4811
Never	479(89.70)	369(89.13)	
Former	17(3.18)	19(4.59)	
Current	38(7.12)	26(6.28)	

Categorical data are presented as percentages (%) and continuous data are presented as mean±standard error (SE). * Significant at *p* < 0.00625 (Bonferroni adjustment).

**Table 2:** Association of exercise and PM_2.5_ with SOX2-promoter methylation in participants.

Variable	β	*p*-Value
Exercise (Ref: No)		
Yes	−0.000538	0.4181
PM_2.5_	0.000216	<0.0001
Sex (Ref: Women)		
Men	0.004940	<0.0001
Age	0.000208	<0.0001
BMI	0.000004	0.9651
Cigarette smoking status (Ref: Never)		
Former	0.001090	0.2553
Current	−0.000789	0.4668
Second-hand smoke exposure (Ref: No)		
Yes	0.000853	0.3847
Alcohol drinking status (ref: Never)		
Former	−0.002490	0.1355
Current	−0.003010	0.0192
Exercise*PM_2.5_	*p*-value = 0.0146	

**Table 3:** Association between PM_2.5_ and SOX2-promoter methylation in participants stratified by exercise habits.

Variable	No Exercise		Exercise	
	β	*p*-Value	β	*p*-Value
PM_2.5_	0.000106	0.1576	0.000349	<0.0001
Sex (Ref: Women)				
Men	0.005010	<0.0001	0.004840	<0.0001
Age	0.000203	<0.0001	0.000220	<0.0001
BMI	−0.000006	0.9577	0.000060	0.6793
Cigarette smoking status (Ref: Never)				
Former	0.002440	0.0737	0.000052	0.9694
Current	0.000160	0.9078	−0.002450	0.1678
Second-hand smoke exposure (Ref: No)				
Yes	0.000604	0.6232	0.001100	0.5107
Alcohol drinking status (ref: Never)				
Former	−0.006370	0.0091	0.001220	0.5906
Current	−0.003700	0.0310	−0.002030	0.2932

**Table 4:** Association of exercise habits and residential area with SOX2-promoter methylation in participants.

Variable	β	*p*-Value
Exercise and PM_2.5_ area (Ref: No exercise in the Northern area)		
No exercise in the North-Central area	0.001100	0.3663
No exercise in the Central area	−0.000913	0.4208
No exercise in the Southern area	0.002610	0.0162
Exercise in the Northern area	−0.003140	0.0036
Exercise in the North-Central area	0.001530	0.2678
Exercise in the Central area	0.001190	0.3595
Exercise in the Southern area	0.002720	0.0172
Sex (Ref: Women)		
Men	0.005050	<0.0001
Age	0.000209	<0.0001
BMI	−0.000034	0.6980
Cigarette smoking status (Ref: Never)		
Former	0.001390	0.1470
Current	−0.000829	0.4415
Second-hand smoke exposure (Ref: No)		
Yes	0.000743	0.4466
Alcohol drinking status (Ref: Never)		
Former	−0.002160	0.1917
Current	−0.003280	0.0104

## Supplementary Materials

The following are available online at https://www.mdpi.com/2072-6694/12/2/504/s1, Table S1: Penalized linear regression (LASSO) showing the association of exercise and PM_2.5_ with SOX2 promoter methylation in participants., Table S2: Penalized linear regression (LASSO) showing the association between PM_2.5_ and SOX2 promoter methylation in participants stratified by exercise habits., Table S3: Penalized linear regression (LASSO) showing the association of exercise habits and residential area with SOX2 promoter methylation in participants.

## Abbreviations

DNA: deoxyribonucleic acid, PM: particulate matter, SHS: second-hand smoke, BMI: body mass index, CpG: cytosine-phosphate-guanine, SE: standard error, β: regression coefficient, n: sample size.

## References

[1] Lovinsky-Desir S., Jung K.H., Jezioro J.R., Torrone D.Z., De Planell-Saguer M., Yan B., Perera F., Rundle A., Perzanowski M., Chillrud S.N. (2017). Physical activity, black carbon exposure, and DNA methylation in the FOXP3 promoter. Clin. Epigenet..

[2] Koutsokera A., Kiagia M., Saif M.W., Souliotis K., Syrigos K. (2013). Nutrition Habits, Physical Activity, and Lung Cancer: An Authoritative Review. Clin. Lung Cancer.

[3] Brown J.C., Winters-Stone K., Lee A., Schmitz K.H. (2012). Cancer, physical activity, and exercise. Compr. Physiol..

[4] Bade B.C., Thomas D.D., Scott J.B., Silvestri G.A. (2015). Increasing Physical Activity and Exercise in Lung Cancer: Reviewing Safety, Benefits, and Application. J. Thorac. Oncol..

[5] Wen C.P., Wai J.P.M., Tsai M.K., Yang Y.C., Cheng T.Y.D., Lee M.-C., Chan H.T., Tsao C.K., Tsai S.P., Wu X. (2011). Minimum amount of physical activity for reduced mortality and extended life expectancy: A prospective cohort study. Lancet.

[6] Arem H., Moore S.C., Park Y., Ballard-Barbash R., Hollenbeck A., Leitzmann M., Matthews C. (2014). Physical activity and cancer-specific mortality in the NIH-AARP Diet and Health Study cohort. Int. J. Cancer.

[7] Michaels C. (2016). The importance of exercise in lung cancer treatment. Transl. Lung Cancer Res..

[8] O’Malley N., Stout B., Wonders K. (2018). The Effects and Efficacy of Exercise in Lung Cancer Patients: An Overview. Health Sci. J..

[9] Schraufnagel D.E., Balmes J.R., Cowl C.T., De Matteis S., Jung S.H., Mortimer K., Perez-Padilla R., Rice M.B., Riojas-Rodriguez H., Sood A. (2018). Air pollution and non-communicable diseases: A review by the forum of international respiratory societies’ environmental committee. Part 2: Air pollution and organ systems. Chest.

[10] Oravisjärvi K., Pietikäinen M., Ruuskanen J., Rautio A., Voutilainen A., Keiski R.L. (2011). Effects of physical activity on the deposition of traffic-related particles into the human lungs in silico. Sci. Total Environ..

[11] Cutrufello P.T., Smoliga J., Rundell K.W. (2012). Small Things Make a Big Difference. Sports Med..

[12] Rundell K.W., Slee J.B., Caviston R., Hollenbach A.M. (2008). Decreased Lung Function After Inhalation of Ultrafine and Fine Particulate Matter During Exercise is Related to Decreased Total Nitrate in Exhaled Breath Condensate. Inhal. Toxicol..

[13] Lovinsky-Desir S., Jung K.H., Rundle A.G., Hoepner L.A., Bautista J.B., Perera F.P., Chillrud S.N., Perzanowski M.S., Miller R.L. (2016). Physical activity, black carbon exposure and airway inflammation in an urban adolescent cohort. Environ. Res..

[14] Sinharay R., Gong J., Barratt B., Ohman-Strickland P., Ernst S., Kelly F.J., Zhang J.J., Collins P., Cullinan P., Chung K.F. (2018). Respiratory and cardiovascular responses to walking down a traffic-polluted road compared with walking in a traffic-free area in participants aged 60 years and older with chronic lung or heart disease and age-matched healthy controls: A randomised, crossover study. Lancet.

[15] IARC (2013). Outdoor Air Pollution a Leading Environmental Cause of Cancer Deaths. http://www.iarc.fr/en/media-centre/pr/2013/pdfs/pr221_E.pdf.

[16] Ahmad Kiadaliri A., Norrving B. (2016). Global, regional, and national comparative risk assessment of 79 behavioural, environmental and occupational, and metabolic risks or clusters of risks, 1990–2015: A systematic analysis for the Global Burden of Disease Study 2015. Lancet.

[17] World Health Organization (2016). Ambient Air Pollution: A Global Assessment of Exposure and Burden of Disease.

[18] Valavanidis A., Fiotakis K., Vlachogianni T. (2008). Airborne Particulate Matter and Human Health: Toxicological Assessment and Importance of Size and Composition of Particles for Oxidative Damage and Carcinogenic Mechanisms. J. Environ. Sci. Health Part C.

[19] Kile M.L., Fang S.C., Baccarelli A.A., Tarantini L., Cavallari J., Christiani D.C. (2013). A panel study of occupational exposure to fine particulate matter and changes in DNA methylation over a single workday and years worked in boilermaker welders. Environ. Health.

[20] Chen G., Wan X., Yang G., Zou X. (2015). Traffic-related air pollution and lung cancer: A meta-analysis. Thorac. Cancer.

[21] Tomczak A., Miller A.B., Weichenthal S.A., To T., Wall C., Van Donkelaar A., Martin R.V., Crouse D.L., Villeneuve P.J. (2016). Long-term exposure to fine particulate matter air pollution and the risk of lung cancer among participants of the Canadian National Breast Screening Study. Int. J. Cancer.

[22] Raaschou-Nielsen O., Andersen Z.J., Beelen R., Samoli E., Stafoggia M., Weinmayr G., Hoffmann B., Fischer P., Nieuwenhuijsen M., Brunekreef B. (2013). Air pollution and lung cancer incidence in 17 European cohorts: Prospective analyses from the European Study of Cohorts for Air Pollution Effects (ESCAPE). Lancet Oncol..

[23] Lo W.-C., Shie R.-H., Chan C.-C., Lin H.-H. (2017). Burden of disease attributable to ambient fine particulate matter exposure in Taiwan. J. Formos. Med. Assoc..

[24] Soberanes S., Gonzalez A., Urich D., Chiarella S., Radigan K.A., Osornio-Vargas A.R., Joseph J., Kalyanaraman B., Ridge K.M., Chandel N.S. (2012). Particulate matter Air Pollution induces hypermethylation of the p16 promoter Via a mitochondrial ROS-JNK-DNMT1 pathway. Sci. Rep..

[25] Wei H., Liang F., Cheng W., Zhou R., Wu X., Feng Y., Wang Y. (2017). The mechanisms for lung cancer risk of PM2.5: Induction of epithelial-mesenchymal transition and cancer stem cell properties in human non-small cell lung cancer cells. Environ. Toxicol..

[26] Cohen A., Brauer M., Burnett R., Anderson H.R., Frostad J., Estep K., Balakrishnan K., Brunekreef B., Dandona L., Dandona R. (2017). Estimates and 25-year trends of the global burden of disease attributable to ambient air pollution: An analysis of data from the Global Burden of Diseases Study 2015. Lancet.

[27] Baccarelli A.A., Wright R.O., Bollati V., Tarantini L., Litonjua A.A., Suh H.H., Zanobetti A., Sparrow D., Vokonas P.S., Schwartz J. (2009). Rapid DNA Methylation Changes after Exposure to Traffic Particles. Am. J. Respir. Crit. Care Med..

[28] Herceg Z., Ghantous A., Wild C.P., Sklias A., Casati L., Duthie S., Fry R.C., Issa J.-P.J., Kellermayer R., Koturbash I. (2018). Roadmap for investigating epigenome deregulation and environmental origins of cancer. Int. J. Cancer.

[29] Martin E.M., Fry R.C. (2018). Environmental Influences on the Epigenome: Exposure- Associated DNA Methylation in Human Populations. Annu. Rev. Public Health.

[30] Wright R.O., Schwartz J., Wright R.J., Bollati V., Tarantini L., Park S.K., Hu H., Sparrow D., Vokonas P., Baccarelli A.A. (2010). Biomarkers of Lead Exposure and DNA Methylation within Retrotransposons. Environ. Health Perspect..

[31] De Prins S., Koppen G., Jacobs G., Dons E., Van De Mieroop E., Nelen V., Fierens F., Panis L.I., De Boever P., Cox B. (2013). Influence of ambient air pollution on global DNA methylation in healthy adults: A seasonal follow-up. Environ. Int..

[32] Bellavia A., Urch B., Speck M., Brook R.D., Scott J., Albetti B., Behbod B., North M., Valeri L., Bertazzi P.A. (2013). DNA Hypomethylation, Ambient Particulate Matter, and Increased Blood Pressure: Findings From Controlled Human Exposure Experiments. J. Am. Hear. Assoc..

[33] Mostafavi N., Vermeulen R., Ghantous A., Hoek G., Probst-Hensch N., Herceg Z., Tarallo S., Naccarati A., Kleinjans J., Imboden M. (2018). Acute changes in DNA methylation in relation to 24 h personal air pollution exposure measurements: A panel study in four European countries. Environ. Int..

[34] Li R., Zhou R., Zhang J. (2018). Function of PM2.5 in the pathogenesis of lung cancer and chronic airway inflammatory diseases. Oncol. Lett..

[35] Weina K., Utikal J. (2014). SOX_2_ and cancer: Current research and its implications in the clinic. Clin. Transl. Med..

[36] Santini R., Pietrobono S., Pandolfi S., Montagnani V., D’Amico M., Penachioni J.Y., Vinci M.C., Borgognoni L., Stecca B. (2014). SOX2 regulates self-renewal and tumorigenicity of human melanoma-initiating cells. Oncogene.

[37] Wong O.G.-W., Huo Z., Siu M.K.-Y., Zhang H., Jiang L., Wong E.S.-Y., Cheung A.N.-Y. (2010). Hypermethylation of SOX_2_ Promoter in Endometrial Carcinogenesis. Obstet. Gynecol. Int..

[38] Yoon H.I., Park K.H., Lee E.-J., Keum K.C., Lee C.G., Kim C.H., Kim Y.B. (2016). Overexpression of SOX_2_ Is Associated with Better Overall Survival in Squamous Cell Lung Cancer Patients Treated with Adjuvant Radiotherapy. Cancer Res. Treat. Off. J. Korean Cancer Assoc..

[39] Maehara R., Fujikura K., Takeuchi K., Akita M., Abe-Suzuki S., Karbanova J., Corbeil D., Itoh T., Kakeji Y., Zen Y. (2018). SOX_2_-silenced squamous cell carcinoma: A highly malignant form of esophageal cancer with SOX2 promoter hypermethylation. Mod. Pathol..

[40] Ren Z.-H., Zhang C.-P., Ji T. (2016). Expression of SOX2 in oral squamous cell carcinoma and the association with lymph node metastasis. Oncol. Lett..

[41] Aydın E.B., Sezgintürk M.K. (2017). A sensitive and disposable electrochemical immunosensor for detection of SOX2, a biomarker of cancer. Talanta.

[42] Ying J., Shi C., Li C.-S., Hu L.-P., Zhang W.-D. (2016). Expression and significance of SOX2 in non-small cell lung carcinoma. Oncol. Lett..

[43] Marycz K., Mierzejewska K., Śmieszek A., Suszyńska E., Malicka I., Kucia M., Ratajczak M.Z. (2016). Endurance Exercise Mobilizes Developmentally Early Stem Cells into Peripheral Blood and Increases Their Number in Bone Marrow: Implications for Tissue Regeneration. Stem Cells Int..

[44] Voisin S., Eynon N., Yan X., Bishop D. (2015). Exercise training and DNA methylation in humans. Acta Physiol..

[45] Horsburgh S., Robson-Ansley P., Adams R., Smith C. (2015). Exercise and inflammation-related epigenetic modifications: Focus on DNA methylation. Exerc. Immunol. Rev..

[46] Tantoh D.M., Wu M.-F., Ho C.-C., Lung C.-C., Lee K.-J., Nfor O.N., Hsu S.-Y., Chen P.-H., Lin C., Chu H.-W. (2019). SOX2 promoter hypermethylation in non-smoking Taiwanese adults residing in air pollution areas. Clin. Epigenet..

[47] Fisher J.E., Loft S., Ulrik C.S., Raaschou-Nielsen O., Hertel O., Tjønneland A., Overvad K., Nieuwenhuijsen M.J., Andersen Z.J. (2016). Physical Activity, Air Pollution, and the Risk of Asthma and Chronic Obstructive Pulmonary Disease. Am. J. Respir. Crit. Care Med..

[48] Tainio M., de Nazelle A.J., Götschi T., Kahlmeier S., Rojas-Rueda D., Nieuwenhuijsen M.J., de Sá T.H., Kelly P., Woodcock J. (2016). Can air pollution negate the health benefits of cycling and walking?. Prev. Med..

[49] Ruiz-Hernández A., Kuo C.-C., Rentero-Garrido P., Tang W.-Y., Redón J., Ordovas J.M., Navas-Acien A., Tellez-Plaza M. (2015). Environmental chemicals and DNA methylation in adults: A systematic review of the epidemiologic evidence. Clin. Epigenet..

[50] Martin E.M., Fry R.C. (2016). A cross-study analysis of prenatal exposures to environmental contaminants and the epigenome: Support for stress-responsive transcription factor occupancy as a mediator of gene-specific CpG methylation patterning. Environ. Epigenet..

[51] Subach O.M., Maltseva D.V., Shastry A., Kolbanovskiy A., Klimašauskas S., Geacintov N.E., Gromova E.S. (2007). The stereochemistry of benzo [a] pyrene-2′-deoxyguanosine adducts affects DNA methylation by SssI and HhaI DNA methyltransferases. FEBS J..

[52] Wang Y., Zhong Y., Hou T., Liao J., Zhang C., Sun C., Wang G. (2019). PM2.5 induces EMT and promotes CSC properties by activating Notch pathway in vivo and vitro. Ecotoxicol. Environ. Saf..

[53] Metz E.P., Rizzino A. (2019). Sox_2_ dosage: A critical determinant in the functions of Sox2 in both normal and tumor cells. J. Cell. Physiol..

[54] Chen S., Xu Y., Chen Y., Li X., Mou W., Wang L., Liu Y., Reisfeld R.A., Xiang R., Lv D. (2012). SOX_2_ Gene Regulates the Transcriptional Network of Oncogenes and Affects Tumorigenesis of Human Lung Cancer Cells. PLoS ONE.

[55] Xiang R., Liao D., Cheng T., Zhou H., Shi Q., Chuang T.S., Markowitz D., Reisfeld R.A., Luo Y. (2011). Downregulation of transcription factor SOX2 in cancer stem cells suppresses growth and metastasis of lung cancer. Br. J. Cancer.

[56] Alonso M.M., Diez-Valle R., Manterola L., Rubio A., Liu D., Cortes-Santiago N., Urquiza L., Jauregi P., De Munain A.L., Sampron N. (2011). Genetic and Epigenetic Modifications of Sox_2_ Contribute to the Invasive Phenotype of Malignant Gliomas. PLoS ONE.

[57] Velcheti V., Schalper K., Yao X., Cheng H., Kocoglu M., Dhodapkar K., Deng Y., Gettinger S., Rimm D.L. (2013). High SOX2 Levels Predict Better Outcome in Non-Small Cell Lung Carcinomas. PLoS ONE.

[58] Wilbertz T., Wagner P., Petersen K., Stiedl A.-C., Scheble V.J., Maier S., Reischl M., Mikut R., Altorki N.K., Moch H. (2011). SOX_2_ gene amplification and protein overexpression are associated with better outcome in squamous cell lung cancer. Mod. Pathol..

[59] Sholl L.M., Barletta J.A., Yeap B.Y., Chirieac L.R., Hornick J.L. (2010). Sox2 protein expression is an independent poor prognostic indicator in stage I lung adenocarcinoma. Am. J. Surg. Pathol..

[60] Brcic L., Sherer C.K., Shuai Y., Hornick J.L., Chirieac L.R., Dacic S. (2012). Morphologic and Clinicopathologic Features of Lung Squamous Cell Carcinomas ExpressingSox_2_. Am. J. Clin. Pathol..

[61] Avinçsal M.O., Jimbo N., Fujikura K., Shinomiya H., Otsuki N., Morimoto K., Furukawa T., Morita N., Maehara R., Itoh T. (2018). Epigenetic down-regulation of SOX2 is an independent poor prognostic factor for hypopharyngeal cancers. Histopathology.

[62] Li X.-L., Eishi Y., Bai Y.-Q., Sakai H., Akiyama Y., Tani M., Takizawa T., Koike M., Yuasa Y. (2004). Expression of the SRY-related HMG box protein SOX2 in human gastric carcinoma. Int. J. Oncol..

[63] Tantoh D.M., Lee K.-J., Nfor O.N., Liaw Y.-C., Lin C., Chu H.-W., Chen P.-H., Hsu S.-Y., Liu W.-H., Ho C.-C. (2019). Methylation at cg05575921 of a smoking-related gene (AHRR) in non-smoking Taiwanese adults residing in areas with different PM2.5 concentrations. Clin. Epigenet..

[64] Fan C.-T., Lin J.-C., Lee C.-H. (2008). Taiwan Biobank: A project aiming to aid Taiwan’s transition into a biomedical island. Pharmacogenomics.

[65] Solomon O., MacIsaac J., Quach H., Tindula G., Kobor M., Huen K., Meaney M.J., Eskenazi B., Barcellos L.F., Holland N. (2018). Comparison of DNA methylation measured by Illumina 450K and EPIC BeadChips in blood of newborns and 14-year-old children. Epigenetics.

[66] Bojovic B., Blancher C. (2017). Epigenetic analysis on Illumina EPIC arrays. Epigenetics.

[67] Rahmani E., Zaitlen N., Baran Y., Eng C., Hu N., Galanter J., Oh S., Burchard E.G., Eskin E., Zou J. (2016). Sparse PCA corrects for cell type heterogeneity in epigenome-wide association studies. Nat. Methods.

